# A quality improvement initiative for patient knowledge comprehension during the discharge procedure using a novel computer-generated patient-tailored discharge document in cardiology

**DOI:** 10.1177/20552076221129079

**Published:** 2022-09-25

**Authors:** André de Wit, John de Heide, Paul Cummins, Ada van Bruchem-van de Scheur, Rohit Bhagwandien, Mattie Lenzen

**Affiliations:** 1 Department of Cardiology, 6993 Erasmus MC, Rotterdam, The Netherlands; 2 6985 Rotterdam University of Applied Sciences, Rotterdam, The Netherlands

**Keywords:** Health informatics, education, lifestyle, cardiology, medicine, electronic, personalized medicine

## Abstract

**Objective:**

The duration of hospital admissions has shortened significantly. This challenges healthcare professionals to provide the necessary information and instructions in a limited time. Patient-tailored discharge information may improve the patient's understanding of the discharge information but may also be time-consuming. The objective of this descriptive quality improvement study was to evaluate patient comprehension of discharge information using a novel computer-generated patient-tailored discharge document.

**Methods:**

A prospective pre-post study comparing patient-tailored discharge information with conventional discharge information, for patients undergoing an electrophysiological procedure during two periods of six weeks between January and March 2016.

Group I received conventional discharge information (*n*  =  55). Group II received a computer-generated, patient-tailored discharge document (*n*  =  57). Their comprehension of the discharge information was evaluated using a peer-reviewed questionnaire distributed among patients, comparing groups I and II using Likert scales. Nurses and nurse practitioners evaluated the use of personalized discharge information by means of a short survey.

**Results:**

In terms of discharge information, comprehensibility was equivalent; however, an increase in comprehension was observed in patients seeking a telephone consultation with the cardiology department within one-week post-discharge. A reduction in discharge preparation time and an increased uniformity of discharge information were reported by nurses. Nurse practitioners found the web tool easy to use and time-saving.

**Conclusions:**

In this study, computer-generated patient-tailored discharge information was equivalent to conventional discharge information. A more positive trend was seen for patients who initiated teleconsultation with the hospital within one-week post-discharge. This suggests that for this subgroup the patient-tailored discharge web tool might lead to an improvement in care. However, more research with a larger number of participants is needed to confirm this trend.

## Introduction and background

Patients require comprehensive information concerning their illness, treatment and daily management, which entails conveying sufficient knowledge to patients by healthcare professionals, with the objective of improving compliance and self-care.^1^ The current duration of hospital admission has shortened significantly,^2^ which challenges healthcare professionals to provide the necessary information and instructions. There is only a limited amount of time available to provide instructions, but also the timing is shortly after an invasive procedure in which patients may be less receptive to receiving information.^3^ The provided instructions should at least cover lifestyle and discharge (aiming at reducing post-procedural complications) since these instructions are important to improve outcomes.^4^ Poor health literacy can result in delayed treatment of post-procedural complications as the potential post-intervention symptoms are not recognized, and thus these patients do not contact a healthcare provider in time.^5^ Importantly, the older the patient the higher the risk of a knowledge deficit which may consequently result in an increased risk of adverse events.^6^

Typically, the essential information is conveyed verbally along with standardized patient discharge information booklets. Although these standardized booklets have been shown to increase patient knowledge retention,^7,8^ it has been advocated that personalized (patient-tailored) discharge information may further improve patient comprehension,^9,10^ especially if it is integrated into a patient-tailored discharge procedure.^11^

The routine practice for patients undergoing invasive electrophysiological procedures at our center includes a standardized discharge information booklet and non-structured verbal post-discharge instructions from a healthcare provider (e.g. nurse(-practitioner) and/or medical doctor). In addition, a telephone follow-up, approximately 1-week post-discharge, is included in the clinical practice. During this follow-up, which includes an evaluation of the hospitalization, it was observed that the discharge instructions provided proved unclear for some patients (particularly for adjustment in medication regimen), consequently leading in some cases to increased patient anxiety. This resulted in an initiative to improve the post-discharge procedure by providing patient-tailored information in a uniform discharge document created using a dedicated web-based tool.

The aim of this pilot study was to evaluate patient knowledge comprehension prior and post introduction of a novel computer-generated patient-tailored discharge document.

## Methods

### Study population

The implementation of a novel computer-generated patient-tailored discharge document was evaluated in this prospective pre-post study performed at our center, a tertiary referral University Hospital in an urban area population of 1,015,000 inhabitants. From January to March 2016, all consecutive patients undergoing an invasive percutaneous diagnostic or therapeutic electrophysiological procedure who fulfilled the study criteria were included in this study. Patients were eligible if they were 18 years or older and fluent both in oral and written Dutch. Exclusion criteria were requiring an additional invasive procedure during the same admission or treatment other than a percutaneous electrophysiological procedure. The control group (group I) received standard discharge information and was included during the first six weeks of the study. The study group (group II) received the novel computer-generated patient-tailored discharge information and document during the second period of six weeks. In both groups, patient relatives were preferably present when the discharge information was conveyed. One week post-discharge a peer-reviewed questionnaire was sent to both groups to evaluate their retention and comprehension of the provided discharge instructions, the clarity of the medication regimen, the recovery process, and finally the overall evaluation of the discharge procedure. To avoid a potential bias between each group, the usual telephonic follow-up, 1 week after discharge, was not carried out during the timeline of this study. For patients who contacted the cardiology ward seeking aid after the ablation, additional specifics were noted as to the reason for the contact and what was done to assist the patients.

### Discharge information

Group I received the standardized discharge information booklet, as well as general verbal instructions, including the advice to consult the booklet in case of uncertainty concerning possible complications.

**Fig. 1.**
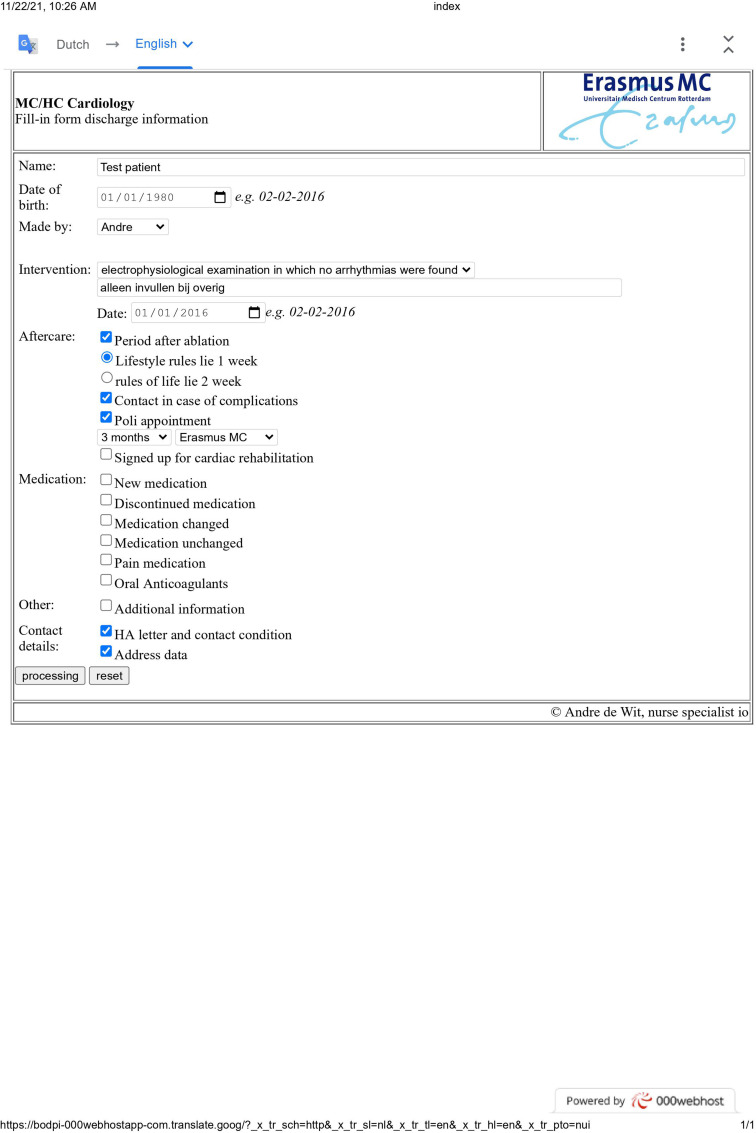

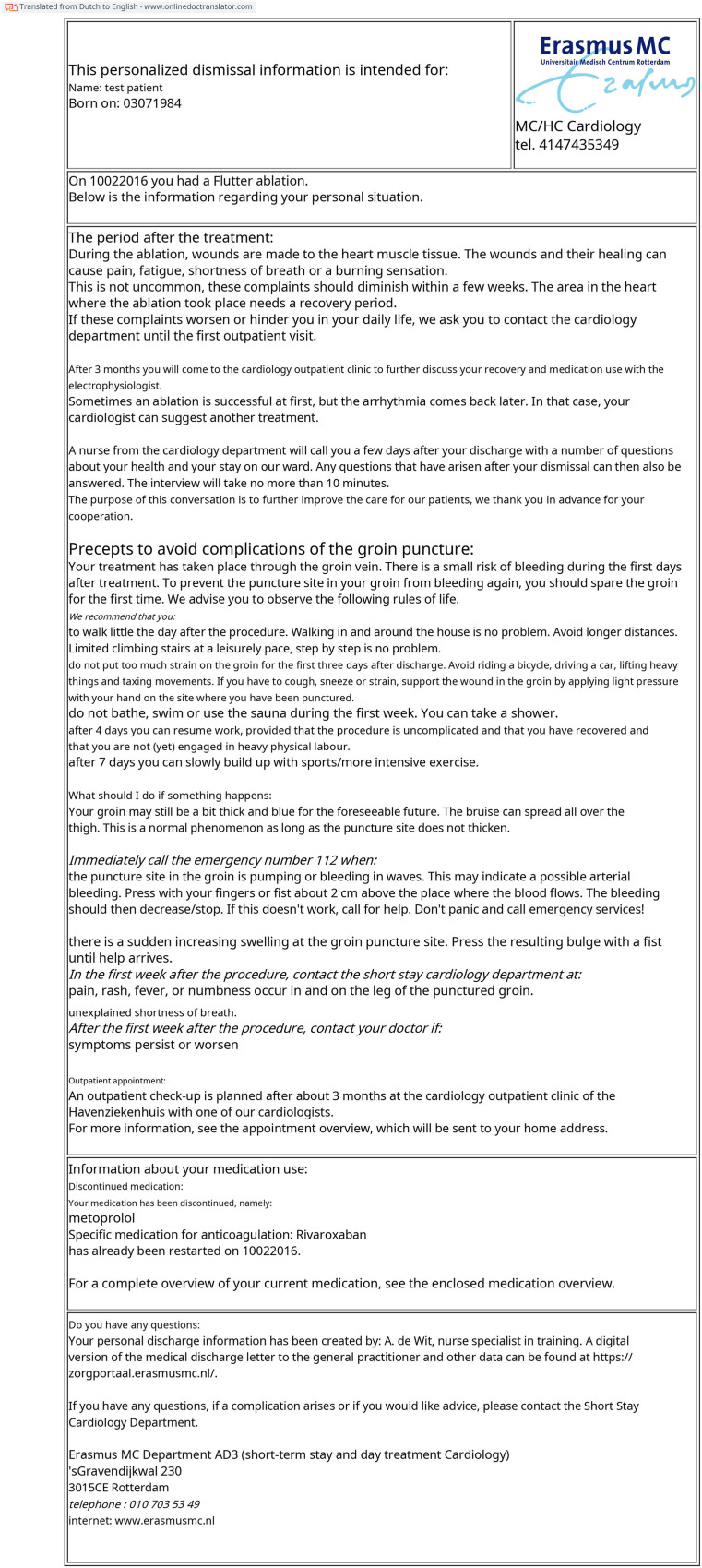
Figure 1a. ICT tool. Figure 1b. Personalized discharge information part 1. Figure 1c—Personalized discharge information part 2.

Group II received the novel patient-tailored discharge information, which included a computer-generated document based on predefined variables (via check-boxes), as illustrated in Figure 1(a). The variables were derived from the analysis of two years of telephone patient follow-up data of 931 patients between 2010 and 2012, including patients discharged after the percutaneous electrophysiological intervention. Also, a small subgroup of 10 patients was surveyed to review an early version of the novel discharge information for lay-out and for the necessity of additional information. Minor alterations were done to the personalized discharge information afterward. The consequences of these various enhancements were that group II received both more specific and for some variables more extensive information than group I.

The variables included clinical and procedural characteristics namely: type of ablation (relevant due to the number and diameter of used catheters), type of percutaneous access (arterial or venous), anticoagulation regimen and post-procedural bleeding or hematoma formation. Additionally, information on discharge medication (and dose), explicitly stating “altered, new or unchanged,” date of the outpatient appointment and whether cardiac rehabilitation is advised, were also provided. If necessary, additional information via free text could be entered. Importantly, a dedicated and secure server within the hospital IT structure was adopted to host the web-based tool. Based on this information, patient-tailored discharge information and instructions, consisting of one or two pages following a predefined format were printed (Figure 1(b)/1(c))). This document was dispensed to patients and used by healthcare providers to convey the discharge information uniformly and clearly, in addition to serving as a reference document for the guidance of patients after discharge.

### Questionnaire

**Fig. 2.**
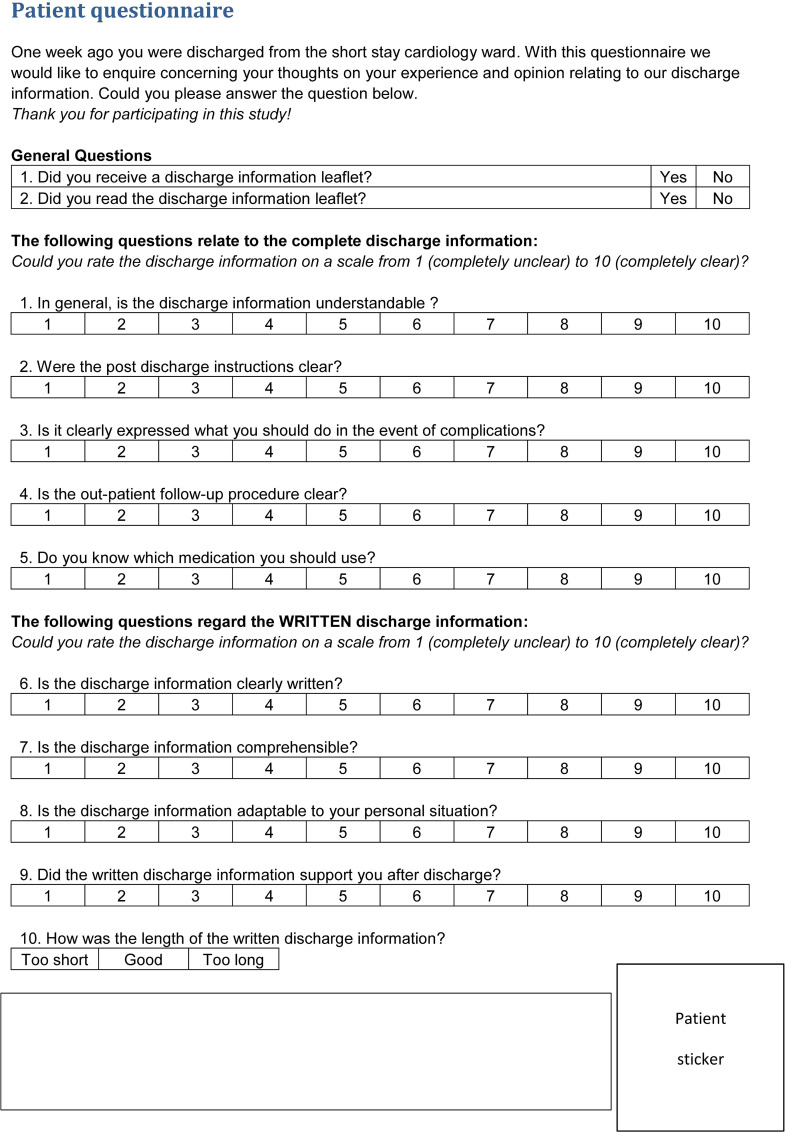
Patient questionnaire.

The peer-reviewed questionnaire was based on the analysis of two years of telephone patient follow-up data of 931 patients between 2010 and 2012, including patients discharged after percutaneous electrophysiological intervention. This analysis revealed poor health literacy, poor retention of (provided) information and associated increased levels of uncertainty among patients. Consequently, a panel of three nurse practitioners, an interventional cardiologist (electrophysiologist) and an epidemiologist developed a questionnaire consisting of 10 questions using a Likert scale from 1 to 10 as depicted in Figure 2.

The questionnaire measured the comprehensibility of the given information by reviewing the responses provided. Patients unable to comprehend the written documents were excluded from the study. Therefore, no levels of comprehension are reported.

**Fig. 3.**
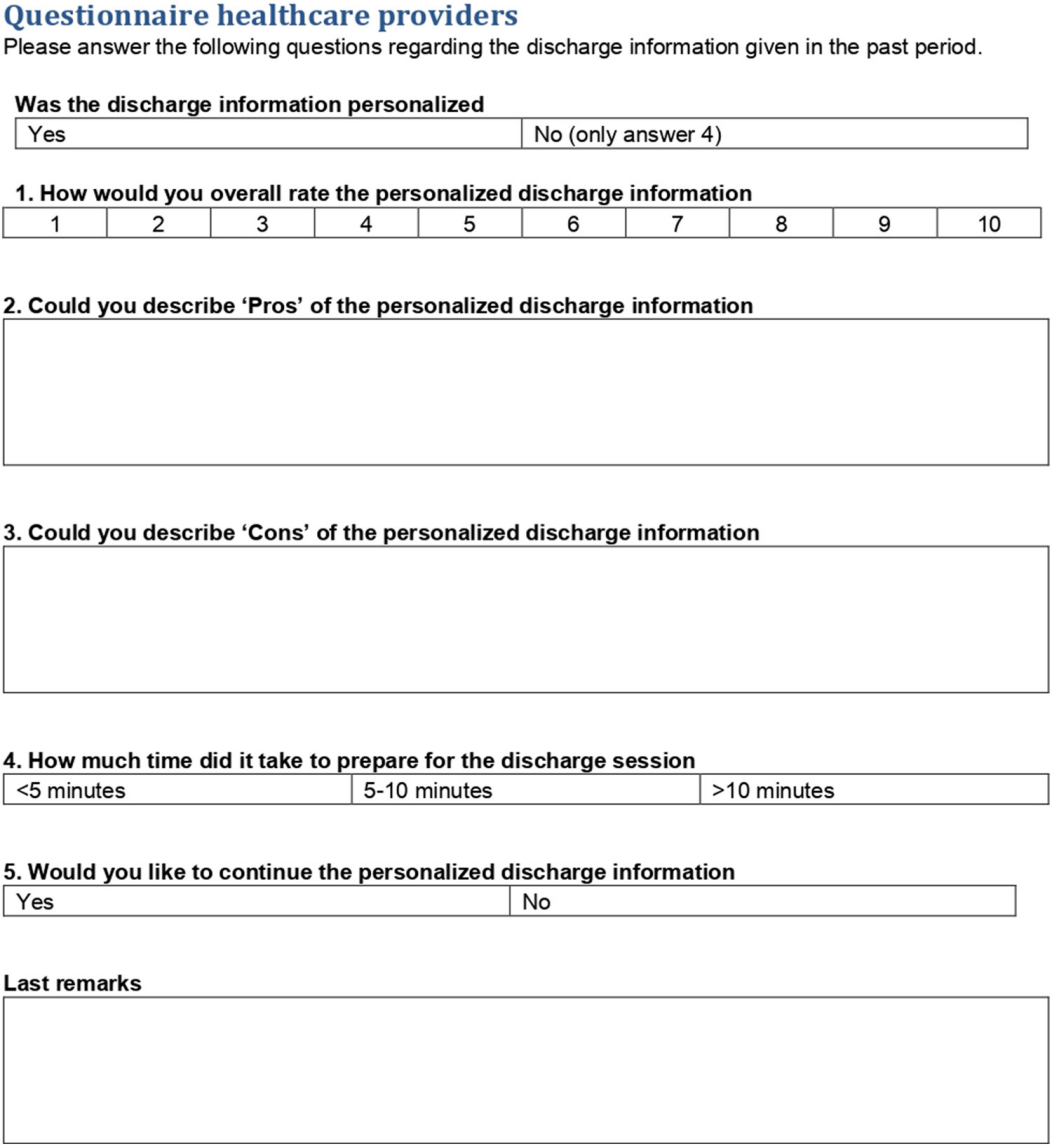
Healthcare provider questionnaire.

As well as sending questionnaires to participants, nurses and nurse practitioners received a short questionnaire to evaluate their experiences with the computer-generated patient-tailored discharge document. This questionnaire consisted of one Likert scale question, one categorized question, two yes/no questions and three open questions as outlined in Figure 3.

The Medical Ethics Committee of our center reviewed the study and deemed the study was not subject to the Dutch Medical Research Involving Human Subjects Act and hence no formal approval was required. The study was conducted in accordance with the Declaration of Helsinki.^12^ All participants provided written consent.

### Study endpoint

The primary endpoint was an improvement in comprehension of the discharge information. Secondary endpoints were usability and feasibility as reported by the nurses and nurse practitioners.

### Data collection and analysis

Baseline characteristics were collected from the electronic patient record. The comprehension of the discharge information (control group vs study group) was measured using the Likert scales in the questionnaire, completed one-week post-discharge and compared using the Student's t-test, Pearson's chi-squared test or Mann–Whitney U test, as deemed appropriate.

In addition, the use of computer-generated discharge information was evaluated among nurse(-practitioners).

Continuous data are presented as mean  ±  SD or median with IQRs and compared between the two groups with the Student's t-test or Mann–Whitney U test, as appropriate. Categorical data are presented as frequencies and percentages and compared with chi-square or the Fisher exact test, as appropriate. Statistical analyses were performed using SPSS software (SPSS, version 25; IBM, Chicago, Illinois).

## Results

**Fig. 4.**
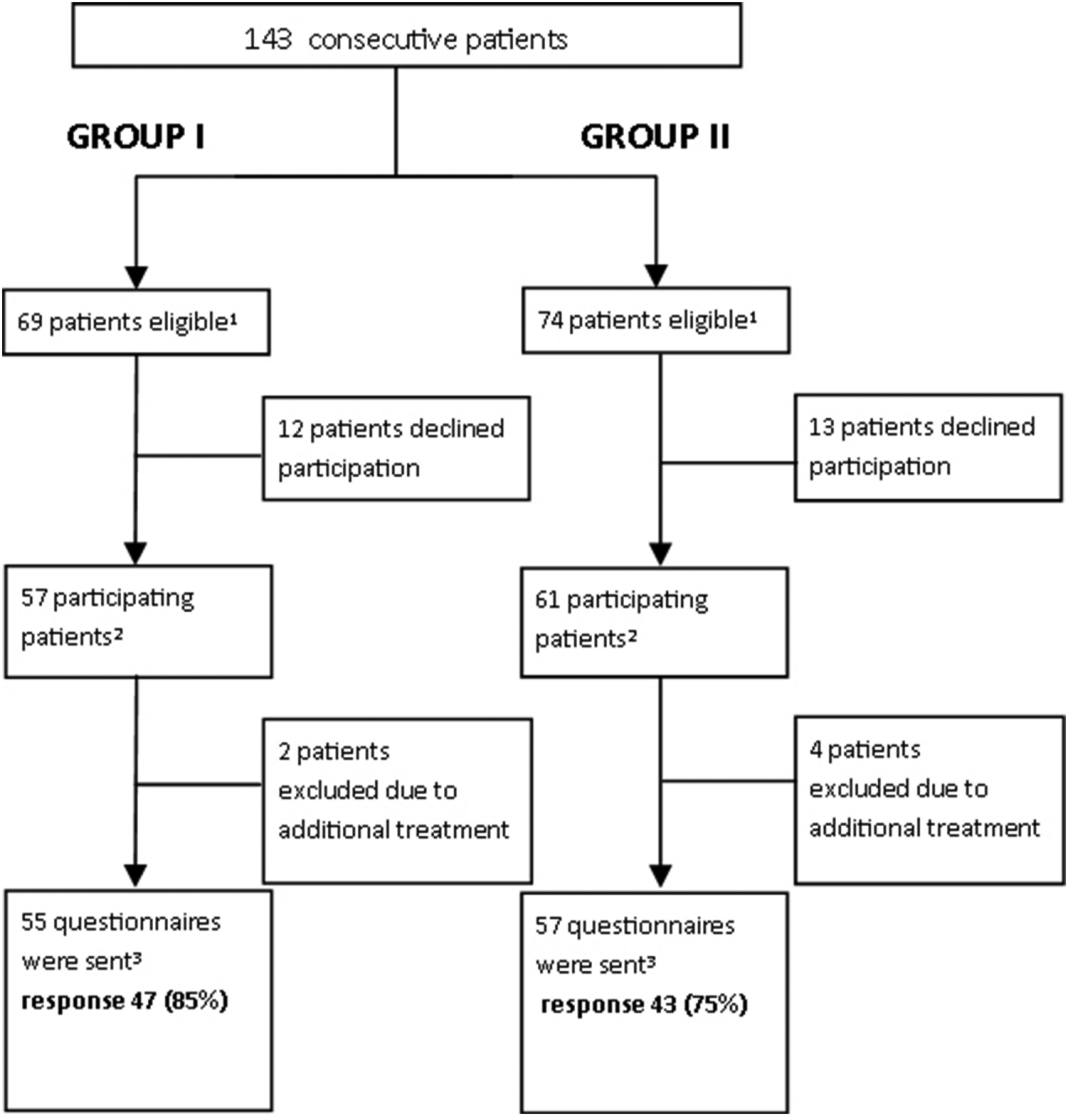
Patient inclusion workflow.

A total of 143 patients were eligible for the participation of whom 31 did not fulfill the study criteria (26 patients did not consent, three patients were excluded due to the need for additional pacemaker implantation or implantable cardiac monitor, and in two patients the procedure was canceled). As a result, the final study cohort consisted of 112 patients (conventional information, *n*  =  55; patient-tailored information, *n*  =  57) as shown in Figure 4. In 100 patients (89%), patient relatives were present during the discharge session. The patient characteristics were comparable between the two groups (Table 1) and ablation for atrial fibrillation (*n*  =  42, 38%) and atrial flutter (*n*  =  17, 15%) were the most prevalent electrophysiological procedures (Table 2). The mean admission time was 1.9 days (± 0.9).

**Table 1.** Patient characteristics and cardiac risk factors.

	Total *n* = 112	Conventional information *n* = 55	Patient-tailored information *n* = 57	*P-*value
Age, mean ± SD	57 ± 13	57 ± 14	57 ± 13	0.94
Male, *n* (%)	58 (52)	30 (55)	28 (49)	0.57
Smoking current, *n* (%)	16 (14)	9 (16)	7 (12)	0.92
BMI, *n* (%)				0.30
Low (<18.5)	2 (2)	2 (4)	0 (0)	
Normal (18.5–25)				
High (>25)	67 (62)	34 (63)	33 (60)	
Diabetes, *n* (%)	4 (4)	2 (4)	2 (4)	0.35
Dyslipidemia, *n* (%)	14 (13)	7 (13)	7 (12)	0.35
Hypertension, *n* (%)	23 (21)	9 (16)	14 (25)	0.22
Family history of cardiac disease, *n* Mann–Whitney U test (%)	27 (24)	17 (30)	10 (18)	0.25

BMI: body mass index; SD: standard deviation.

Significant if *P*-value ≤ 0.05, determined with Pearson's chi-squared test.

**Table 2.** Performed percutaneous electrophysiological procedures.

		**Total** *n* = 112	Conventional information *n* = 55	Patient-tailored information *n* = 57	*P-*value
Electrophysiology study (no ablation performed)		11 (10)	6 (11)	5 (9)	0.70
Ablation (defined per type)	Left-sided accessory pathway	6 (5)	3 (6)	3 (5)	0.96
	Right-sided accessory pathway	2 (2)	1 (2)	1 (2)	0.98
	Atrial-ventricular re-entry tachycardia	14 (13)	6 (11)	8 (14)	0.62
	Atrial tachycardia	1 (1)	0 (0)	1 (2)	0.32
	Atrial flutter	17 (15)	9 (16)	8 (14)	0.93
	Atrium fibrillation	42 (38)	21 (38)	21 (37)	0.73
	Premature ventricular complex	11 (10)	6 (11)	5 (9)	0.71
	Ventricular tachycardia	6 (5)	2 (4)	4 (7)	0.42
	His bundle	2 (2)	1 (2)	1 (2)	0.98

All data is depicted as *n* (%).

Significant if *P*-value ≤ 0.05, determined with Pearson's chi-squared test.

### Discharge information

A total of 90 questionnaires (80% response) assessing the comprehension of discharge information as reviewed by patients were returned. The overall discharge information scored 8.6 (±1.2) for group I (conventional information) and 8.8 (±1.0) for group II (patient-tailored information) on the 10-point Likert scale, with high scores (>8.5) in all subcategories (Table 3).

**Table 3.** Outcome patient questionnaire.

	**Group I** **mean (±SD)**	**Group II** **mean (±SD)**	**Absolute difference**	***P*-value**
	***(n* *=* *47, 85% response)***	***(n* *=* *45, 79% response)***	*** ***	*** ***
***Response on the clarity of the information presented at the discharge talk***				
Overall clarity	8.6 (1.2)	8.8 (1.0)	+ 0.2	0.55
Post discharge instructions	8.8 (1.2)	8.9 (1.0)	+ 0.1	0.53
Possible complications	8.7 (1.2)	8.9 (1.1)	+ 0.2	0.43
Expected recovery process	8.6 (1.4)	8.7 (1.2)	+ 0.1	0.74
New or altered medication	9.0 (1.3)	8.9 (1.1)	−0.1	0.61
***Response on the clarity of the way the discharge information was written***				
Clearly written	8.8 (1.0)	8.7 (1.0)	−0.1	0.73
Comprehensible	8.9 (1.0)	8.7 (1.0)	−0.2	0.58
Specific for the patient	8.6 (1.3)	8.7 (1.4)	+ 0.1	0.82
Did discharge information help after discharge	8.4 (1,5)	8,4 (1,3)	0	0.87

Significant if *P*-value ≤ 0.05, determined with Pearson's chi-squared test.

Of the patients who contacted the cardiology department within one week after discharge (*n* = 12, 11%), 9 patients returned the questionnaire. In this particular subgroup, the differences between the conventional and patient-tailored discharge information (8.0 ( ± 0.9) vs 9.2 ( ± 1.2)) showed a greater improvement in comprehension, favoring group II. The most important reasons for contacting the cardiology department included groin/leg complaints, palpitations, dizziness, or concerns about the prescribed medication. Seven of these patients (*n* = 3, group I; *n* = 4, group II) were evaluated at the emergency department, none of whom required a re-admission.

### Survey among healthcare professionals

The survey among those who provided the discharge information (registered nurses, *n*  =  8) and those who prepared the discharge information (nurse practitioners, *n*  =  3), expressed a preference for the novel patient-tailored discharge information documents compared to the conventional booklet information. Registered nurses gave the personalized discharge information an 8.1  ±  0.84 on a scale from 0 to 10 and nurse practitioners gave it a 7.7  ±  0.58 (Table 4). Both nurses and nurse practitioners remarked that the personalized discharge information should be extended into daily practice, chiefly because of the personalized nature, improved consistency of the provided information and that all information was consolidated in one document. In addition, it was also reported by nurses that the novel procedure required less time for preparing and providing the actual discharge information. The reason for this improvement in time-saving was that all the information regarding medication changes, outpatient clinic appointments, etc. was easily included in the personalized discharge information document and did not, therefore, need to be extracted from different sources such as the ward secretary or nurse practitioner. The personalized discharge information is created by the nurse practitioner. This requires more time in composition. However, the nurse practitioners noted that this increase is compensated by the convenience of the ICT tool and that the information was already available. Furthermore, improved uniformity was specified as an improvement of the patient-tailored discharge procedure.

**Table 4.** Outcome healthcare provider questionnaire.

	**Nurses**	**Nurse practitioners**
	*(n* *=* *8)*	*(n* *=* *3)*
Overall clarity mean (SD)	8.4 (0.8)	7.1 (0.6)
Time taken		NA
Traditional discharge info		
<5 min (n, %)	1, 12%	
5–10 min (n, %)	7, 88%	
>10 min (n, %)	0, 0%	
Personalized discharge info		
<5 min (n, %)	7, 88%	
5–10 min (n, %)	1, 12%	
>10 min (n, %)	0, 0%	
Would like to continue personalized discharge info (n, %)	8, 100	3, 100

## Discussion

This pilot study evaluated the implementation of a novel discharge procedure, based on a computer-generated patient-tailored document. Importantly, both groups (pre- and post-implementation) evaluated the discharge procedure as favorable and comparable, with scores higher than 8.5. Consequently, no further improvement of the provided information may be required. However, a small subgroup of patients, those who initiated teleconsultation with the department after discharge, showed a trend towards increased comprehension of the provided discharge instructions. Although this small group does not reach scientific significance, it is considered by the authors as a harbinger that personalized discharge information can be an effective solution to increase the quality of post-discharge care. Additionally, healthcare professionals (e.g. nurses involved in the discharge procedure) reported a decrease in time needed for preparing and providing discharge information while maintaining the quality of the delivered information and enhancing uniformity in discharge information.

In contrast to previous literature which reported that patients often feel unprepared for hospital discharge due to a lack of information.^5,6^ This study demonstrated that the discharge information (before and after the implementation of patient-tailored information) satisfied patient expectations resulting in high evaluation scores. The necessity of personalization and preparation is also shown by Kang^13^ and Rushton.^14^ Both these studies verified a lack of preparedness at discharge but also indicated an increase in preparedness when using personalized information.

Naturally, one can question, aside from socially desirable responses on the part of the patients, whether the applied questionnaires (using a Likert scale from 1 to 10) were the best way to evaluate patient satisfaction and the comprehensibility of the provided discharge information. Moreover, the questions posed were elementary, unambiguous, and based on routine standard follow-up questions. Open-ended questions may have provided more detailed responses concerning information comprehension and could be considered in future studies. Additionally, the conventional discharge information also used some form of written procedural information, which increases patient preparedness.^7,8^ In contrast to previous reports, in which patient comprehension increased when using patient-tailored discharge information, this could not be replicated in the current pilot study.^9,10^ One of the reasons might be the difference in comprehension assessment between the studies. While Lin^9^ used a telephonic follow-up where a physician scored patient understanding, Bench^10^ used a peer-reviewed questionnaire with closed questions. Due to the allotted timeframe of our study, a telephonic follow-up was not possible. After e-mail contact with Bench, their questionnaires were reviewed for our needs but did not include the discharge sections we wanted to address, probably because of the difference in a clinical setting (ICU vs cardiac short stay). For this reason, we developed and used our own peer-reviewed questionnaire using a Likert scale.

When focusing on the small subgroup of patients who contacted the department for consultation, those who received patient-tailored information conveyed the impression of an improved level of knowledge. This may indicate that patient-tailored discharge information could have resulted in a lower threshold to actively contact the hospital for consultation. It should be noted, however, that this aspect falls out of the scope of this study and could be included in future studies, for instance, with a specific qualitative research design employing interviews to ensure detailed analysis.

Creating personalized discharge information, as reported in the literature,^9^ is typically reviewed as too time-consuming for use in a clinical setting, specifically when this is handwritten. The application of this computer tool as evaluated in the current study proved to be both convenient and time-saving, while providing adequate information to patients. Moreover, this procedure has been demonstrated to be a feasible alternative in generating patient-tailored discharge documents and consequently has been now fully implemented in our department.

Limitations of the current study include the single center character of the study, the small sample size, and the use of non-validated questionnaires. Also, one may consider the influence of patient relatives who were present during the discharge sessions, in this study 89%. These effects are not evaluated in this study.

Future research is warranted to optimize the discharge process. The current personalization of discharge information appears to improve the discharge process. More information is however necessary which could not be derived from the current study. A mixed method study using questionnaires, open-ended questions and interviews could provide more insights concerning gaps in information and the reasons why patients act as they do. This information could be used in an early stage to optimize the discharge information.^15^

## Conclusion

In this pilot study a novel discharge procedure, using patient-tailored discharge information, was demonstrated to be equivalent to conventional discharge information, and was evaluated as easier to use. A positive trend was observed for patients who initiated teleconsultation with a healthcare question after discharge. This may suggest that for this subgroup the patient-tailored discharge tool can lead to lowering the threshold to contact healthcare providers and consequently leading to improvements in care. However, further research is warranted to better evaluate this effect.
